# Male asymptomatic hyperuricemia patients display a lower number of NKG2D^+^ NK cells before and after a low-purine diet

**DOI:** 10.1097/MD.0000000000013668

**Published:** 2018-12-14

**Authors:** Lichao Gao, Yanfang Jiang, Yichen Wang, Xiaozhang Qu, Lei Li, Xiaoqian Lou, Ye Wang, Hui Guo, Ya Liu

**Affiliations:** a The School of Public Health; b Department of Endocrinology of The First Hospital, Jilin University; c Genetic Diagnosis Center; d Key Laboratory of Zoonoses Research, Ministry of Education, The First Hospital of Jilin University, Changchun; e Jiangsu Co-Innovation Center for Prevention and Control of Important Animal Infectious Diseases and Zoonoses, Yangzhou, China.

**Keywords:** asymptomatic hyperuricemia, BMI, NK cells, NKG2D, SUA

## Abstract

**Background:**

Aberrant activation of the immune system has been reported in asymptomatic hyperuricemia (HUA) patients. However, very few studies have elucidated the role of natural killer (NK) cells in this disease.

**Methods:**

In this study, we evaluated the relationship between NK cells and HUA in 16 control subjects and 20 patients, who were all on a low-purine diet. We analyzed the number of circulating NK cells, its subsets, interferon-γ^+^, and CD107^+^ NK cells, by flow cytometry, before and after 4 and 24 weeks of diet control. We also assessed the potential association of the NK cells with clinical measures.

**Results:**

The patients consistently had a lower number of NKG2D^+^ NK cells before and after low-purine diet, even the serum uric acid (SUA) levels <7 mg/dL after diet control. Moreover, a lower number of NK cells and a higher number of CD107a^+^ NK cells were observed on recruitment. Low-purine diet was benefit on the improvement of the SUA levels, body mass index (BMI), and the number and functions of NK cells. Furthermore, the number of CD3^−^CD56^+^ NK cells and NKG2D^+^ NK cells negatively correlated with the BMI before and after diet control.

**Conclusion:**

The consistent lower number of NKG2D^+^ NK cells and correlated with BMI before and after low-purine diet may be involved in the occurrence and development of HUA.

## Abstract 2

Supplemental Digital Content is available in the text

## 1 Introduction

Uric acid (UA), the end product of purine metabolism in the human body, plays a pro-oxidative or an antioxidative role in different pathophysiological environments.^[1]^ Its accumulation in the blood is termed as hyperuricemia, and its incidence was increased largely in the last few years.^[2,3]^ Asymptomatic hyperuricemia (HUA) is typically associated with a disease called gout, which is characterized by the deposition of monosodium urate (MSU) crystals in the joint and soft tissues. However, multiple reports also have indicated that HUA is closely correlated with many other diseases, such as type 2 diabetes mellitus^[4]^ and peripheral artery disease.^[5]^ The lowering of UA levels in these patients has resulted in improvement in insulin resistance^[6]^ and the glomerular filtration rate.^[7]^ Despite HUA playing a pivotal role in many disease processes, there has been little consensus about its management.

Studies focusing on the mechanism of HUA have indicated that its occurrence is due to either excessive production and/or decreased elimination of serum uric acid (SUA); however, the exact pathophysiological mechanism is not fully understood. In recent years, many additional studies had discovered a close relationship between SUA levels and the immune system.^[8–11]^ The UA levels in the body have been shown to be associated with an increased IgG1-based humoral immunity response,^[8]^ in addition to its effects on Th17 cell differentiation,^[9]^ thereby indicating a role for UA in the adaptive immune response. Moreover, soluble UA or MSU crystals seem to act as an endogenous danger signal and can trigger natural immunity. In one study, the authors observed that healthy peripheral blood mononuclear cells (PBMCs) stimulated with UA increased the levels of pro-inflammatory cytokines and reduced the levels of anti-inflammatory cytokines.^[10]^ Systemic inflammation also has been observed in patients with HUA.^[11–13]^ In addition, these patients showed elevated serum levels of monocyte chemotactic protein-1 (MCP-1) along with an increased percentage of circulating CD14^+^ monocytes.^[11]^ Elevated SUA levels are strongly associated with C-reactive protein (CRP) levels as well as the serum levels of various cytokines, including interleukin (IL)-6, IL-1ra, and IL-18.^[12]^ In comparison to adults, the SUA concentration in children has been shown to be less affected by diet, alcohol intake, and diseases. The study by Wasilewska et al has reported that children with HUA had elevated levels of serum MCP-1 and high-sensitivity CRP.^[13]^ The UA-lowering treatments have resulted in improvement of the overall systemic inflammation.^[6]^ The MSU crystals have been shown to trigger gouty arthritis by regulating the functions of macrophages and neutrophils, and the drugs that improve inflammation have been beneficial to patients with gout.^[14]^ Similarly, MSU crystal deposition also has been detected in the joints of hyperuricemic patients,^[15]^ indicating the possible activation of local macrophages and neutrophils. In addition, another study has demonstrated the expansion of CD56^bright^ natural killer (NK) cells in the joints of gout patients.^[16]^ However, so far, no association between NK cells and HUA has been suggested, despite NK cells being an important part of the innate immune system.

Human NK cells are classically defined by the presence of CD56 and the absence of CD3 proteins on the cell surface and can be further divided into different subpopulations based on the relative expression of the surface marker proteins CD56 and CD16. The 2 major subsets are CD56^dim^ and CD56^bright^, respectively.^[17]^ In addition, NK cells express a series of different activating and inhibitory receptors.^[18]^ In humans, the inhibitory receptors include the family of killer cell Ig-like receptors and the lectin-like heterodimer CD94-NKG2A,^[19]^ while activating receptors that stimulate their cytotoxicity include NKG2C, NKG2D, NKP30, NKP46, and NKP44.^[20]^ Overall, the fine balance between the activating and inhibitory receptors on NK cells controls their activity,^[20]^ and many studies have implicated these NK cells in the pathogenesis of various metabolic diseases, such as diabetes^[21]^ and obesity.^[22]^ However, the role of NK cells in the pathogenesis of HUA still needs to be explored.

Therefore, in this study, we investigated the association between HUA and NK cells by studying the alteration of the absolute numbers and functions of NK cells in the peripheral blood of HUA patients and control subjects (CS), before and after 4 and 24 weeks of a low-purine diet. Furthermore, we assessed the expression of activating and inhibitory receptors on the surface of CD3^−^CD56^+^ NK cells. At last, we also assessed the potential association of the NK cells with clinical measures.

## 2 Materials and methods

### 2.1 Selection of study subjects

A total of 20 HUA patients were recruited from the Outpatient Department of The First Hospital of Jilin University, Changchun, China, between May 2015 and January 2016. Because of the estrogen affect the SUA metabolism and different diagnosis criteria of female from male,^[3]^ we selected male patients as objects to exclude the affection of these factors. All patients were either newly diagnosed or diagnosed previously but had not received any treatment. Also, in parallel, another 16 gender-, age-, and ethnicity-matched CS were recruited from the Physical Examination Center of our hospital during the same period. The diagnosis criteria for hyperuricemia included an SUA level above 7 mg/dL.^[2]^ In addition, patients and CS diagnosed with autoimmune diseases such as type 1 diabetes or rheumatoid arthritis, who suffered from chronic inflammatory diseases (such as inflammatory bowel disease) or chronic renal disease, or had taken medicine (such as thiazide diuretics) to regulate SUA levels were excluded from this study. All subjects were given a low-purine diet, which consisted of reduced alcohol intake, avoidance of eating seafood, drinking more water, and adequate intake of vegetables and fruits. None of these subjects accepted UA-lowering drugs during the study period. The demographic and clinical data of individual participants were collected from the hospital records and were reviewed by endocrinologists. The complete records were available for 15 patients, and 5 patients failed to be followed up and thus had partial information. This study was approved by the Institutional Ethics Board at the School of Medicine, Jilin University. In addition, prior to enrollment in the study, all subjects were provided with a clear explanation of the objectives and implications of the study, and all participants signed the consent forms.

### 2.2 Collection of blood samples

Fasting venous blood samples were collected from all participants in the morning (after at least 8 hours of fasting) for routine laboratory analysis, according to standard techniques. Ten milliliters of venous blood was collected in heparinized tubes for immuno-phenotyping of lymphocytes by flow cytometry.

### 2.3 Immuno-phenotyping and measurement of IFN-γ production and NK cell degranulation

PBMCs were isolated from the blood drawn from the subjects by density-gradient centrifugation using the Ficoll-Paque Plus method (Amersham Biosciences, Little Chalfont, UK). These isolated PBMCs (10^6^ cells/well) were then stimulated with 2 μL of Leukocyte Activation Cocktail containing BD GolgiPlug protein transport inhibitor (BD Biosciences, San Jose) in complete RPMI 1640 medium (Invitrogen, Carlsbad, CA) and incubated with 5 μL of phycoerythrin (PE)-Cy5-anti-CD107α antibody (BD Biosciences) in 5% CO_2_ at 37°C for 1 hour. Next, they were further incubated with 1 μL of GolgiStop Protein Transport Inhibitor (BD Biosciences) for an additional 3 hours. Subsequently, the nonadherent cells were harvested, washed with cold phosphate-buffered saline, and stained with PE-Cy7-anti-CD3, APC-HC-anti-CD16, BV510-anti-CD56, PE-CF594-anti-NKG2D, BB515-anti-NKP46, PE-anti-CD158b (BD Biosciences), and APC-anti-NKG2A (R&D, Minnesota) antibodies in the dark at room temperature for 25 minutes. Later, after washing, the cells were fixed and permeabilized using a fixation/permeabilization kit (BD Biosciences). Subsequently, intracellular staining with 2 μL of BV421-anti-IFN-γ antibody (BD Biosciences) was performed, and then the cells were analyzed by flow cytometry. In parallel, negative control cells were stained with isotype-matched control antibodies (PE-Cy7-anti-IgG1, APC-HC-anti-IgG_2A_, BV510-anti-IgG_2b_, PE-anti-IgG_2b_, PE-CF594-anti-IgG1, BB515-anti-IgG1, PE-Cy5.5-anti-IgG1, FITC-anti-IgG2a, APC-anti-IgG1, BV421-anti-IgG1, and PE-Cy5-anti-IgG1; BD Biosciences). The percentages of different subsets of NK cells were characterized by a FACSAria II (Becton Dickinson), and at least 500,000 events were analyzed by FlowJo software (v5.7.2, TreeStar Inc, Ashland, OR). The cell number of each subset was calculated according to the percentages of the specific cell type multiplied by the lymphocyte count.

### 2.4 Statistical analyses

All data were expressed as median (range), actual number or percentage of cases. Comparisons between the groups were tested by 1-way analysis of variance. The difference between the hyperuricemia and CS groups was analyzed by the Mann–Whitney *U* nonparametric test. The differences between the disease onset and post-treatment groups were assessed by the Kruskal–Wallis *H* nonparametric test. The correlation between variables was evaluated by the Spearman rank correlation test using SPSS 19.0 software for Windows (SPSS Inc, Chicago, IL). A 2-sided *P* value of <.05 was considered statistically significant.

## 3 Results

### 3.1 Low-purine diet partially reduced SUA levels and BMI

The clinical characteristics displayed by the HUA patients and the CS were analyzed as shown in Table 1. Compared with CS, the patients had an increased body mass index (BMI) and a higher level of SUA, fasting plasma glucose (FPG), triglyceride (TG), and cholesterol (CHO). After diet control, there was a significant reduction of the concentration of SUA and BMI (Table 2), but still quite high in comparison to the CS. In order to assess the effect of the low purine diet on lowing SUA better, we divided the patients into 3 groups according to the level of SUA: <7.0, 7.0 to 7.9, and ≥8.0 mg/dL. Diet control was benefit for the decline of SUA, but there was still a half of patients in the state of hyperuricemia. Despite no consensus on the usage of SUA-lowering drugs in HUA patients, our results indicated that the lowering of SUA levels with drugs should be considered in patients in whom the SUA level does not match the ideal concentration, even after diet control. Unfortunately, we found the low-purine diet only had a little effect on the improvement of FPG, TG, and CHO.

**Table 1:**
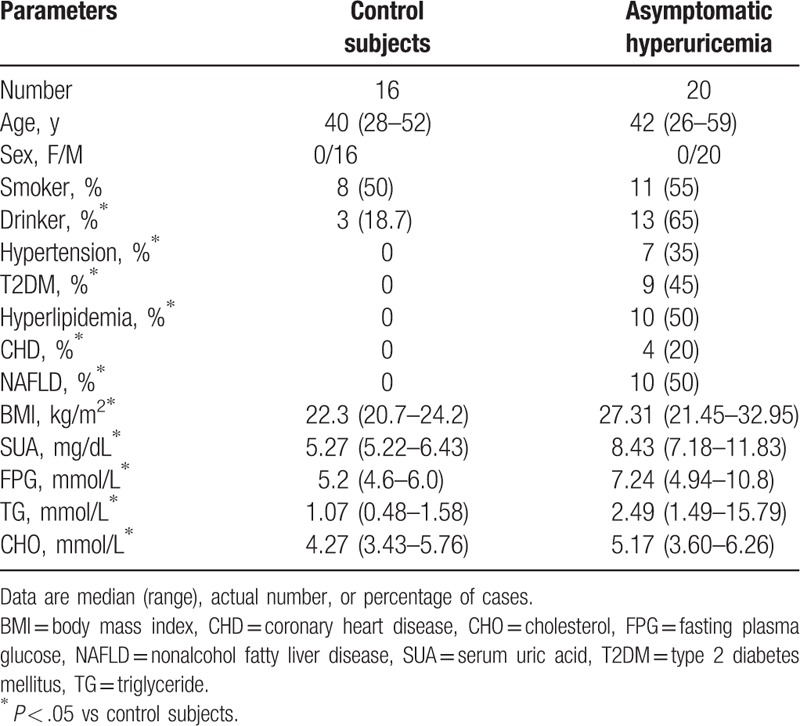
The demographic and clinical characteristics of participants in this study.

**Table 2:**
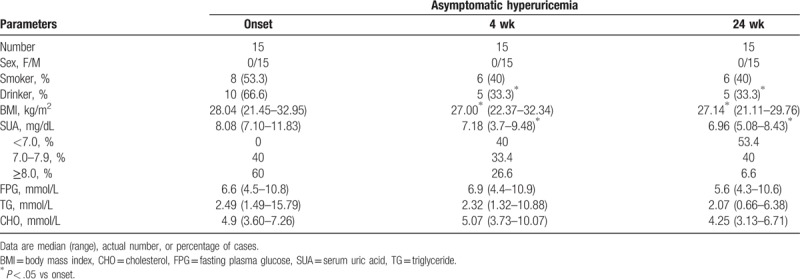
Effects of low purine diet on metabolic parameters.

### 3.2 NKG2D^+^ NK cell population was consistently low in HUA patients before and after a low-purine diet

**Figure 1:**
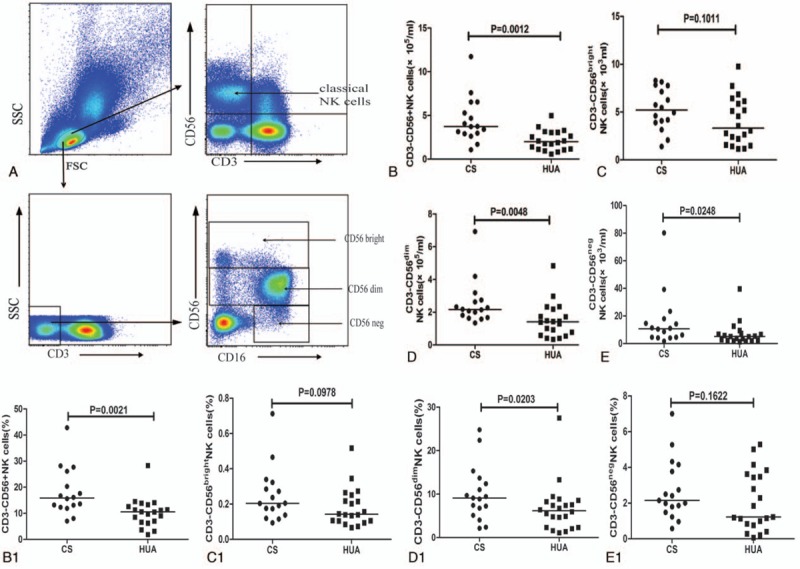
Flow cytometric analysis of NK cells on recruitment. PBMCs from asymptomatic hyperuricemia patients (HUA, n = 20) and control subjects (CS, n = 16) were isolated and stained in duplicate with FITC-anti-CD3, APC-anti-CD56, and PerCP-anti-CD16 antibodies. Panel A represents the flow cytometric strategy. The first set of cells was gated based on CD3 expression. The CD3^−^ cells were further gated based on CD16 and CD56 expression by flow cytometry. Similarly, the second set of cells was gated based on CD3 and CD56 expression by flow cytometry. The absolute numbers (quantitative analysis) and frequency of classical CD3^−^CD56^+^ (B, B1), CD3^−^CD56^bright^ (C, C1), CD3^−^CD56^dim^ (D, D1), and CD3^−^CD56^neg^ (E, E1) NK cells were determined by flow cytometry in samples from all 4 groups, and data were presented as graphs showing the absolute number values. The difference between the groups was analyzed by the Mann–Whitney *U* nonparametric test. The horizontal lines indicate median values. PBMC = peripheral blood mononuclear cell, NK = natural killer.

To understand the correlation of NK cells with the pathogenesis of HUA, we compared different subsets of NK cells at onset of the disease and after diet control (4 and 24 weeks). Flow cytometric analysis indicated that the absolute numbers of CD3^−^CD56^+^ (*P* = .0012), CD3^−^CD56^dim^ NK cells (*P* = .0048), and CD3^−^CD56^neg^NK (*P* = .0248) cells in the peripheral blood were significantly lower in the untreated HUA patients in comparison to the CS, while CD3^−^CD56^bright^ showed no significant difference (Fig. 1). The frequency of CD3^−^CD56^+^ NK cells, CD3^−^CD56^dim^ NK cells, CD3^−^CD56^dim^ NK cells, and CD3^−^CD56^neg^NK cells had the similar alteration with the absolute number (Fig. 1B1–E1). However, 4 weeks of diet control did not alter the absolute numbers of CD3^−^CD56^+^ NK cells in these patients, and they were still lower than those in CS. But interestingly, 24 weeks of diet control resulted in an increase in the absolute numbers of CD3^−^CD56^+^ NK cells in these patients (Fig. 4A), and similar to the numbers in CS (data not shown).

**Figure 2:**
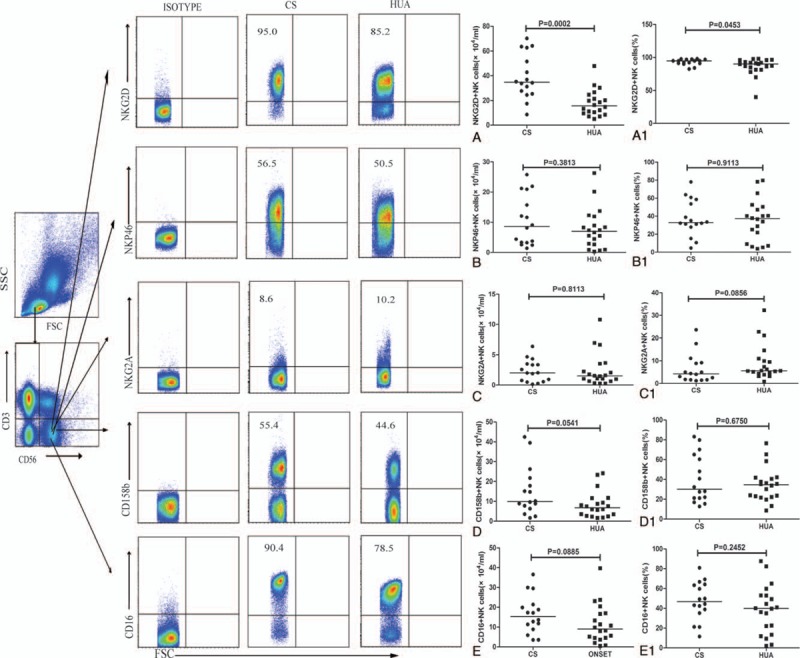
Characterization of different subsets of NK cells on recruitment. PBMCs from hyperuricemia patients (HUA, n = 20) and control subjects (CS, n = 16) were isolated and stained in duplicate with the following antibodies: FITC-anti-CD3, APC-anti-CD56, PerCP-anti-CD16, anti-NKG2D, anti-NKP46, anti-NKG2A, anti-CD158b, and control mouse IgG1 and IgG2a, respectively. Later, the cells were gated based on CD3 and CD56 expression, and CD3^−^CD56^+^ cells were further gated based on NKG2D, NKP46, CD16, NKG2A, and CD158b protein surface expression by flow cytometry. Data are shown as representative graphs expressing individual absolute number values. Panels A–E and A1–E1 represent the quantitative analysis and the frequency of NKG2D^+^ NK cells, NKP46^+^ NK cells, NKG2A^+^ NK cells, CD158b^+^ NK cells, and CD16^+^ NK cells, respectively. The difference between the groups was analyzed by the Mann–Whitney *U* nonparametric test. The horizontal lines indicate median values. PBMC = peripheral blood mononuclear cell, NK = natural killer.

Further analysis of CD3^−^CD56^+^ NK cells based on their activating or inhibitory receptors, like NKG2D, NKp46, NKG2A, and CD158b, indicated a significant decrease in the frequency and absolute number of only NK cells with NKG2D^+^ in HUA patients in comparison to CS (*P* = .0453; *P* = .0002) (Fig. 2A1 and 2A). Moreover, diet control of 4 and 24 weeks did not alter their numbers (Fig. 4B) and were still less than those of CS (data not shown). However, the frequency and absolute numbers of NKP46^+^, NKG2A^+^, CD158b^+^, and CD16^+^ NK cells in HUA patients showed no difference in comparison to CS (Fig. 2B1–E1 and B–E) or even after diet control (Fig. 4C–F).

In addition, we also analyzed the alteration of CD3^−^CD56^dim^ NK cells, CD56^bright^ NK cells, and CD56^neg^ NK cells before and after diet control. Our results indicated a lower number of CD3^−^CD56^dim^ NK cells 4 weeks later, but no significance after 24 weeks when compared with CS. A consistent lower number of NKG2D^+^ CD3^−^CD56^dim^ NK cells also were detected before and after diet control, but there were no difference of the other receptors of CD3^−^CD56^dim^ NK cells (Supplemental Fig. 1-A1). In addition, there was no difference of the receptors of CD56^bright^ (Supplemental Fig. 1B2–G2) and CD56^neg^ subsets (Supplemental Fig. 1B3–G3).

### 3.3 NK cell cytotoxicity increased at onset of HUA but decreased after a low-purine diet

**Figure 3:**
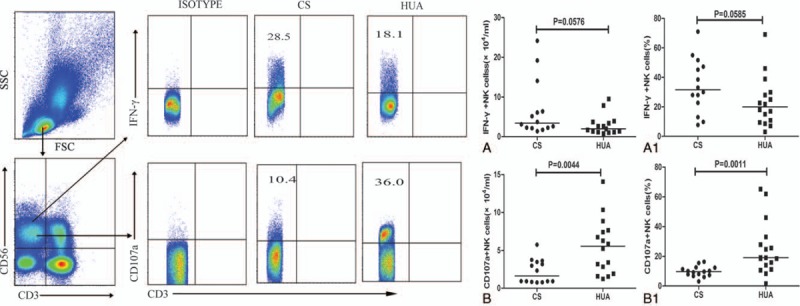
Characterization of inducible IFN-γ^+^ or CD107a^+^ NK cells on recruitment. PBMCs from asymptomatic hyperuricemia patients (HUA, n = 16) and control subjects (CS, n = 14), stimulated with PMA/ionomycin for 4 hours in the presence of PE-Cy5-anti-CD107a antibody, were stained with FITC-anti-CD3, APC-anti-CD56, PerCP-anti-CD16, anti-NKG2D, anti-NKP46, anti-NKG2A, and anti-CD158b. Next, the cells were fixed, permeabilized, and stained intracellularly with BV421-anti-IFN-γ antibody. The absolute numbers and frequency of IFN-γ^+^CD3^−^CD56^+^ NK cells (A, A1) and CD107a^+^ CD3^−^CD56^+^ NK cells (B, B1) were determined by flow cytometry. The difference between the groups was analyzed by the Mann–Whitney *U* nonparametric test. The horizontal lines indicate median values. PBMC = peripheral blood mononuclear cell, PMA = phorbol 12-myristate 13-acetate, NK = natural killer.

**Figure 4:**
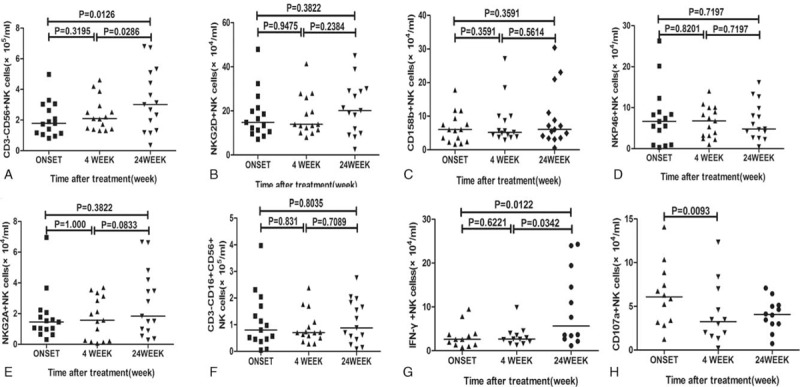
Characterization of NK cells after 4 and 24 weeks low-purine diet. PBMCs from different time of asymptomatic hyperuricemia patients (onset, 4 weeks of diet control, and 24 weeks of diet control, n = 15), stimulated with PMA/ionomycin for 4 hours in the presence of PE-Cy5-anti-CD107a antibody, were stained with FITC-anti-CD3, APC-anti-CD56, PerCP-anti-CD16, anti-NKG2D, anti-NKP46, anti-NKG2A, and anti-CD158b. Next, the cells were fixed, permeabilized, and stained intracellularly with BV421-anti-IFN-γ antibody. Panels A–H represent the quantitative analysis of CD3^−^CD56^+^ NK cells, NKG2D^+^ NK cells, CD158b^+^ NK cells, NKP46^+^ NK cells, NKG2A^+^ NK cells, CD16^+^ NK cells, IFN-γ^+^CD3^−^CD56^+^ NK cells, and CD107a^+^ CD3^−^CD56^+^ NK cells, respectively. The difference between the groups was analyzed by the Wilcoxon test. The horizontal lines indicate median values. PBMC = peripheral blood mononuclear cell, PMA = phorbol 12-myristate 13-acetate, NK = natural killer.

Next, we evaluated the NK cell cytotoxicity in HUA patients by a widely used CD107a degranulation assay. Recently, stimulation of NK cells was achieved with phorbol 12-myristate 13-acetate (PMA) and ionomycin in the CD107a degranulation assay, and a similar effect as the coculture assay with K562 cells was observed.^[23]^ In our study, we used the Leukocyte Activation Cocktail (containing PMA and ionomycin) to stimulate NK cells to acquire IFN-γ and CD107a expression. Our analysis demonstrated that the frequency and absolute numbers of CD107a-secreting NK cells in the patients were significantly higher than those in the CS group (*P* = .0011; *P* = .0044) (Fig. 3B1 and B), and decreased largely after diet control (Fig. 4H). Analyses of the expression of IFN and CD107a on NK cell subsets indicated that the number of NKG2D^+^CD107a^+^ NK cells was increased (Supplemental Fig. 1). By contrast, the IFN-γ-secreting NK cell population was slightly decreased in HUA patients in comparison to the CS (Fig. 3A and A1), and the level was maintained even after 4 weeks of diet control (Fig. 4G).

### 3.4 CD3^−^CD56^+^ NK cells and NKG2D^+^ NK cells showed a negative correlation with BMI in patients with HUA, before and after a low-purine diet

**Figure 5:**
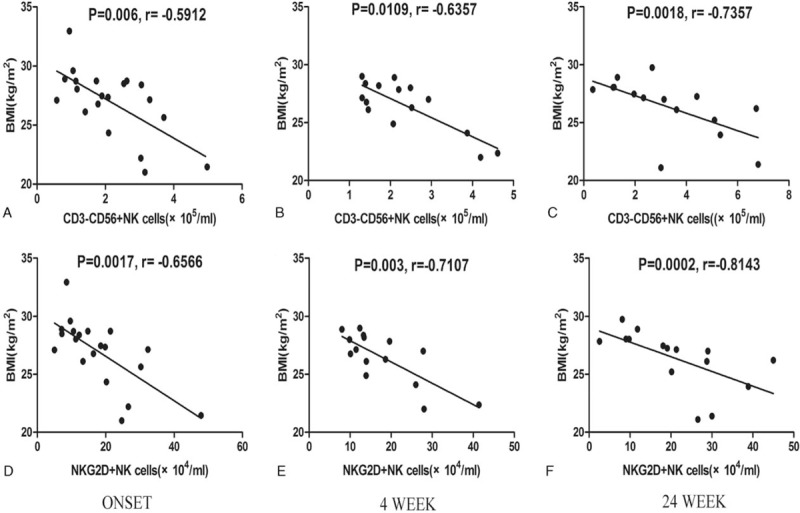
Correlation analysis of NK cells with clinical parameters of asymptomatic hyperuricemia patients. Panels A–C depict the correlation between the absolute number of CD3^−^CD56^+^ NK cells and the body mass index (BMI) before and after 4- and 24-week low-purine diet, while Panels D–F depict the correlation between NKG2D^+^ NK cells and BMI before and after a low-purine diet (n = 15). The individual values of the BMI were plotted against the number of NKG2D^+^ NK cells or CD3^−^CD56^+^ NK cells, and the potential correlation was assessed using Spearman rank correlation test. NK = natural killer.

In addition, we analyzed the pathophysiological association of NK cells with HUA by assessing the correlation between different subsets of circulating CD3^−^CD56^+^ NK cells and clinical parameters. Our results displayed that the absolute number of CD3^−^CD56^+^ NK cells and NKG2D^+^ NK cells was negatively correlated with the BMI values before and after a low-purine diet (Fig. 5). However, we did not observe any correlation between the NK cells with the SUA level before or after a low-purine diet. There was also no correlation between CD3^−^CD56^dim^ NK cells, CD3^−^CD56^bright^ NK cells, CD3^−^CD56^neg^ NK cells, and clinical parameters.

### 3.5 Subgroup analysis indicated a significant decreased activity and increased cytotoxicity before and after low-purine diet in patients with SUA ≥8 mg/dL

**Figure 6:**
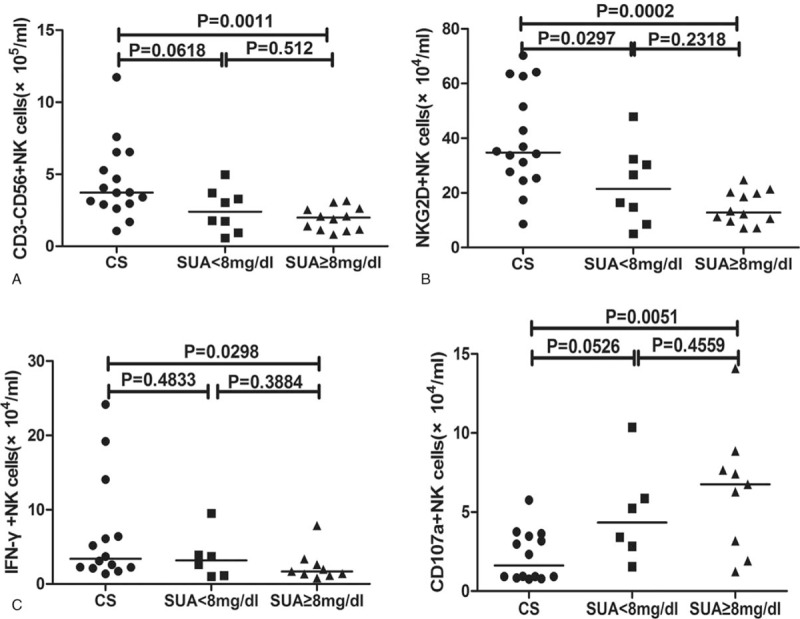
Subgroup analysis of asymptomatic hyperuricemia patients according to the SUA levels on recruitment. The patients (n = 20) were divided into 2 groups according to the SUA levels: <8 and ≥8 mg/dL group. The absolute numbers of CD3^−^CD56^+^ NK cells (A), NKG2D^+^ NK cells (B), IFN-γ^+^CD3^−^CD56^+^ NK cells (C), and CD107a^+^ CD3^−^CD56^+^ NK cells (D) were determined by flow cytometry. The difference between the groups was analyzed by the Mann–Whitney *U* nonparametric test. The horizontal lines indicate median values. NK = natural killer, SUA = serum uric acid.

In order to evaluate the effect of SUA levels on the alteration of NK cells, we divided the onset patients into 2 groups according to the SUA levels: <8 and ≥8 mg/dL groups. Our results indicated that the decreased number of NK cells and NKG2D^+^ NK cells mainly existed in patients with SUA ≥8 mg/dL (Fig. 6A and B). The increased number of CD107a-secreting NK cells and the decreased number of IFN-γ-secreting NK cells were also more obvious in SUA ≥8 mg/dL group (Fig. 6C and D). After the diet control, the alteration of NK cells in patients with SUA ≥8 mg/dL was similar to the whole patients, but there was a mild increase of the numbers of NKG2D^+^ NK cells when compared with onset and 4 weeks (data not shown).

### 3.6 The number of NKG2D^+^ NK cell population was still low in HUA patients when SUA ≤7 mg/dL after diet control

**Figure 7:**
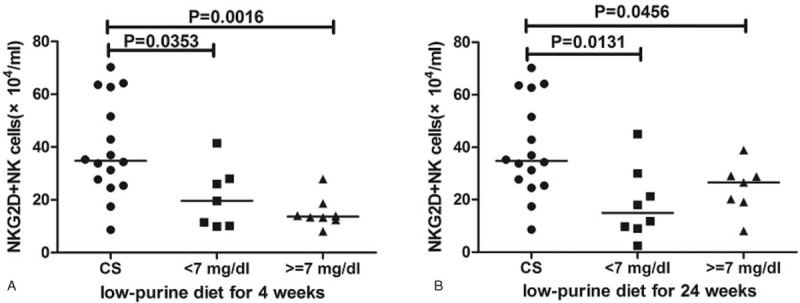
Subgroup analysis of NKG2D^+^ NK cells according to the SUA level after diet control. The patients (n = 15) were divided into 2 groups according to the SUA levels: <7 and ≥7 mg/dL group after 4 and 24 weeks diet control. The absolute numbers of NKG2D^+^ NK cells were determined by flow cytometry on 4 weeks (A) and 24 weeks (B). The difference between the groups was analyzed by the Mann–Whitney *U* nonparametric test. The horizontal lines indicate median values. NK = natural killer, SUA = serum uric acid.

Finally, the SUA levels decreased largely after low-purine diet, so we compared the difference between patients with SUA ≤ 7 mg/dL and CS to evaluate the causation between the NK cells and HUA. Our results indicated that though the SUA level lower than 7 mg/dL, but the number of NKG2D^+^ NK cells was still lower than CS (Fig. 7), but the absolute number of NK cells, NKG2A^+^, NKP46^+^, CD16^+^, CD158b^+^, IFN-γ-secreting and CD107a-secreting NK cells was no difference from CS (data not shown).

## 4 Discussion

We observed that there was a significant decline in the frequency and absolute number of peripheral blood CD3^−^CD56^+^ NK cells and NKG2D^+^ NK cells accompanied by an increased frequency and number of CD107a-secreting NK cells in patients with HUA, especially in patients with SUA ≥8.0 mg/dL. Furthermore, after 4 weeks of a low-purine diet, these patients had decreased SUA levels and BMI, accompanied the decreased number of CD107a-secreting NK cells. Moreover, 24 weeks of diet control had no additional decrease in the SUA levels and BMI, but the number of CD3^−^CD56^+^ NK cells, and NKG2D^+^ NK cells were elevated significantly, at the same time. Our observation implies that the lowering of SUA levels might associated with the restoration of NK cells. Also, the lowering of UA levels (especially when SUA ≥8.0 mg/dL) seems important for effective immune regulation in patients with HUA. There was no consensus on the usage of SUA-lowering drugs in HUA patients, our results indicated that the lowering of SUA levels with drugs should be considered in HUA patients when the SUA levels higher than 8 mg/dL.

NK cell activity, such as cytokine secretion and direct cellular cytotoxicity, is usually a function of a balance between the activating and inhibitory receptors on their surface.^[24]^ Analysis in HUA patients showed a significant decrease in the absolute number of NKG2D^+^ NK cells, especially when the SUA ≥8.0 mg/dL, this phenomenon was more obvious. Diet control had a benefit on the increase expression of the NKG2D. These findings indicate that hyperuricemia have an important influence on the expression of NKG2D. NKG2D is a highly evolutionary and conservative activating receptor on the surface of NK cells, and it recognizes an array of diverse ligands (NKG2DLs) encoded by numerous genes, such as MICA, MICB, and RAET1.^[25,26]^ The binding of NKG2D receptor with its ligands (NKG2DLs) can trigger lysis of the target cell and result in the production of specific cytokines and chemokines, thus regulating the immune response and maintaining tissue homeostasis. Generally, NKG2DLs are absent or have very little expression on the cell surface under normal physiological conditions, but can be induced by various factors, such as tumors, stress, infection, and DNA damage.^[25]^ Multiple studies have shown that NKG2D plays a selective role in different inflammatory and autoimmune diseases.^[27]^ As SUA is mainly produced as a result of DNA degradation, either physiological or pathological, it can act as an endogenous danger signal that causes cell injury and inflammation, such as damage to renal cells through regulation of the nuclear factor-κB signaling pathway^[28]^ and injury to islet β cells by activating the AMPK and ERK signaling pathways.^[29]^ These pathways can also result in induction of NKG2DL on the surface of injured cells, which can subsequently lead to reduced NKG2D expression on the cell surface due to its endocytosis and degradation.^[30]^ In addition, the higher the UA level, the higher the incidence of gout. Monocytes and neutrophils are key immune cells that participate in the initial phase of gouty arthritis, and NK cells have the ability to activate these cells indirectly either through the interaction between them or secreted cytokines.^[31,32]^ Thus, reduced expression of NKG2D indicates a lower activation state of NK cells. In other words, reduced activation of NK cells may avoid a gout attack in patients with HUA especially in patients with SUA ≥8.0 mg/dL. Furtherly, T cells also play an important role in the development of gout and hyperuricemia. As far as we know, NKG2D also expressed on CD8^+^ and γδ^+^T cells, so if the expression of NKG2D on these cells is also decreased need to be explored in the future. It is important to mention here that this is the first longitudinal follow-up study to compare the alteration of the receptors on the NK cell surface in HUA patients before and after a low-purine diet.

To our surprise, the number of NKG2D^+^ NK cells was still lower than CS even the SUA level <7 mg/dL (the cutpoint for hyperuricemia). So, we speculate maybe other factors related with the alteration of NKG2D^+^ NK cells expect for the SUA. In our study, we observed a negative correlation between the number of NK cells, NKG2D^+^ NK cells, and BMI before and after diet control. About 40% our patients were obese and 35% patients were overweight, during follow-up, the SUA, BMI decreased and the number of NK cells and NKG2D^+^ NK cells elevated. In this context, the correlation between BMI and the number of NK cells and NKG2D^+^ NK cells seems to have an important meaning in HUA patients. Findings suggested that adipose tissue can produce and secret UA, this ability might be enhanced during obesity,^[33]^ and the weight-loss lead to the decrease of SUA.^[34]^ In addition, the adipose tissue seems to have infiltration of many immune cells,^[35]^ and multiple studies have indicated the close relationship between obesity and NK cells.^[20,36,37]^ The obese patients displayed a low absolute number of NK cells.^[36]^ NK cells can be found in the circulation and several tissues, including adipose tissue, and the NK cells from different tissues have different functions. Compared with lean subjects, obese subjects tend to have elevated expression of NKG2D on the surface of adipose tissue NK cells.^[37]^ In our study, the NKG2D receptor on circulating NK cells was decreased, but the expression of NKG2D on surface of adipose tissue NK cells was unknown. In brief, adipose tissues may act as a main site for the association of NK cells (especially NKG2D^+^ NK cells) and HUA, but the exact pathway involved needs to be explored in future studies. For the first time, our study identified an association between NK cells, NKG2D^+^ NK cells, and BMI in HUA patients.

In order to exclude the influence of BMI on NK cells, we also detected the alteration of NK cells in another CS (non-HUA) who has the same BMI but without HUA (Supplemental Fig. 2). Compared with the non-HUA group, the HUA patients still had a lower absolute number of CD3^−^CD56^+^ NK cells, NKG2D^+^ NK cells, and a higher absolute number of CD107a^+^ NK cells. That means the lower absolute number of NK cells and decreased expression of NKG2D in HUA patients was a unique phenomenon.

The CD107a degranulation assay is now widely used to assess the antitumor ability of NK cells. Though the number of NK cells and NKG2D^+^ NK cells decreased, our results indicated that the ability of NK cells to secrete IFN-γ was not significantly decreased, and the ability to secrete CD107a was increased. The relationship between serum UA and cancer is not still clear because of the limited studies. Many studies have used MSU crystals as an adjuvant to enhance the antitumor activity of tumor vaccines.^[38,39]^ We speculate that the increased number of CD107a positive cells in hyperuricemia patients maybe an assistant for the MSU crystals’ antitumor ability. That to say, the elevated secretion of CD107a means that NK cells may have protective effect in HUA patients.

In conclusion, our study demonstrated that there is a decreased absolute number of CD3^−^CD56^+^ and NKG2D^+^ NK cells accompanied by an increased number of CD107a-secreting NK cells in patients with HUA, mainly in patients with SUA ≥8.0 mg/dL. In addition, the absolute number of NKG2D^+^ NK cells and NK cells negatively correlated with the BMI. Low-purine diet benefits on the improvement of SUA levels, BMI, and NK cell's number, but the NKG2D^+^ NK cells consistent lower than CS even SUA <7 mg/dL. Thus, we conclude that the consistent lower number of NKG2D^+^ NK cells before and after diet control might have an important role in the pathogenesis of HUA in Chinese male patients, and the main mechanism maybe through adipose tissue. However, owing to the small sample size and gender bias of our study, we propose that an additional study with a larger sample size and conclude female is required to validate our findings. Moreover, in future studies, the effect of drugs for urate-lowering and weight loss on the alteration of NK cell receptors, especially NKG2D, should be explored.

## Acknowledgments

The authors thank Weiwei Cui and Fang Fang for providing help of Statistical analysis. The authors also thank the subjects for their participation in the study.

## Author contributions

**Data curation:** Lei Li.

**Formal analysis:** Ye Wang.

**Investigation:** Ya Liu.

**Methodology:** Yanfang Jiang.

**Project administration:** Xiaoqian Lou.

**Resources:** Xiaozhang Qu.

**Validation:** Yichen Wang.

**Writing – original draft:** Lichao Gao.

**Writing – review & editing:** Hui Guo, Ya Liu.

## Supplementary Material

Supplemental Digital Content
