# Supplementary figures and images for: Male asymptomatic hyperuricemia patients display a lower number of NKG2D+ NK cells before and after a low-purine diet
Source: Medicine (Baltimore). 2018 Dec 14;97(50):e13668. doi: 10.1097/MD.0000000000013668 (PMC6320027; doi:10.1097/MD.0000000000013668)

A1

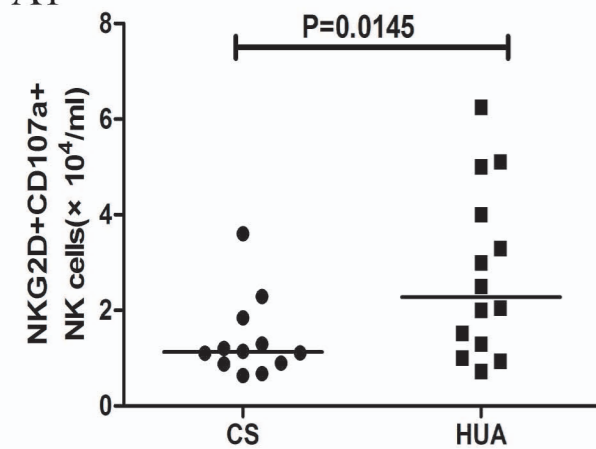

B1

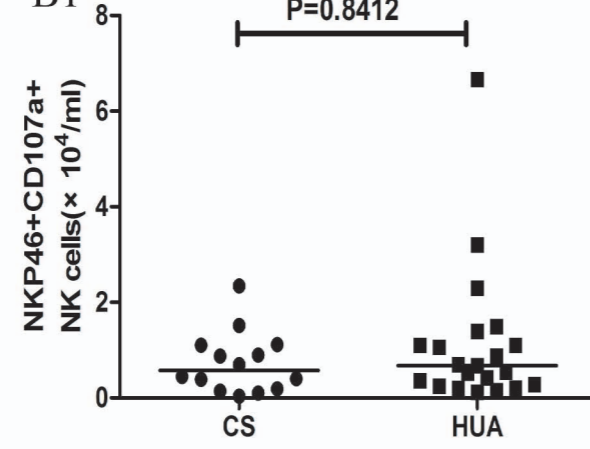

C1

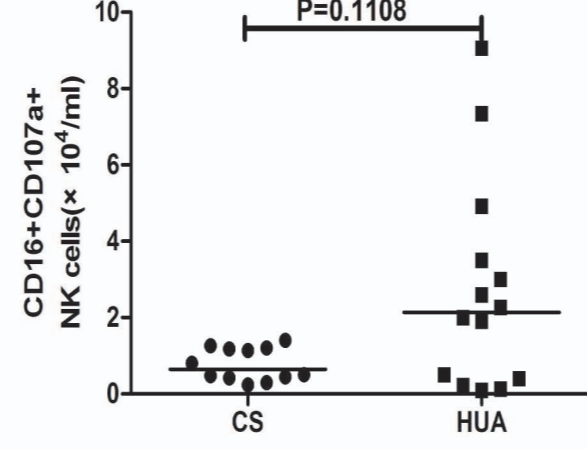

D1

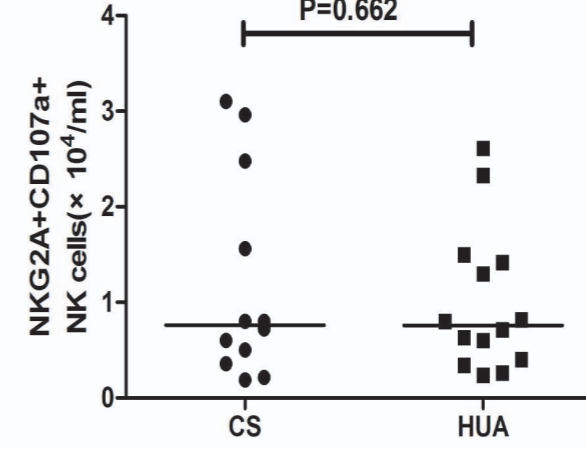

E1

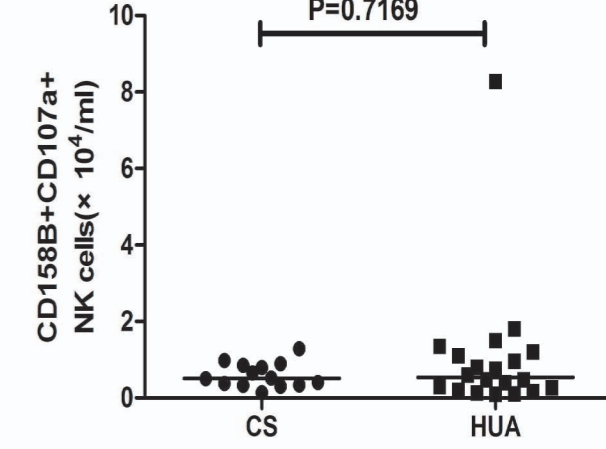

A2

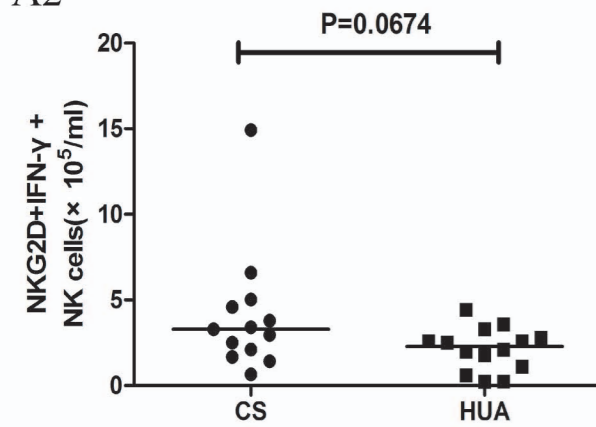

B2

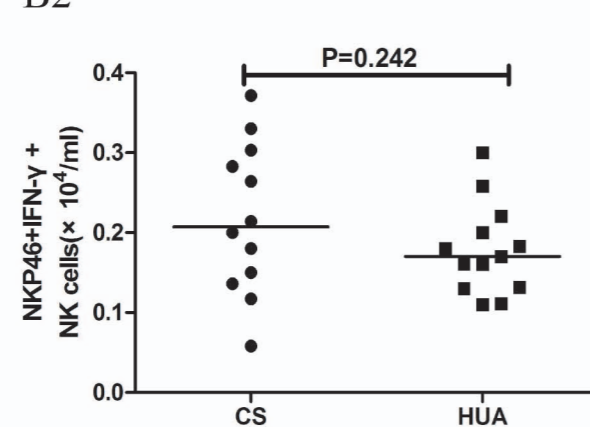

C2

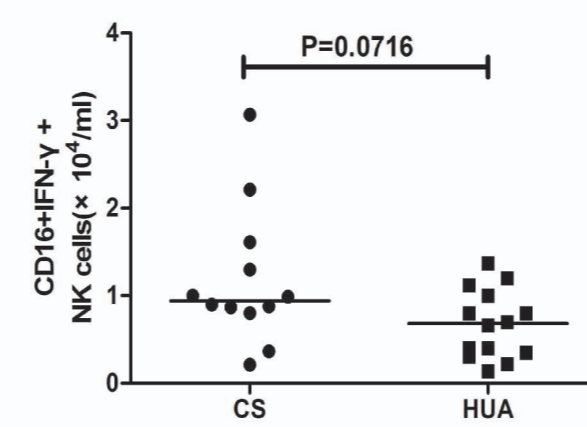

D2

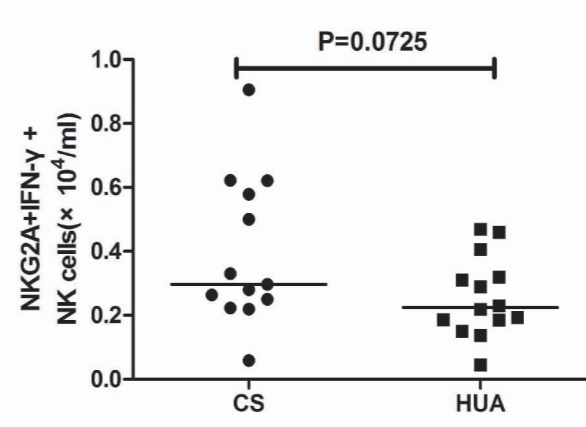

E2

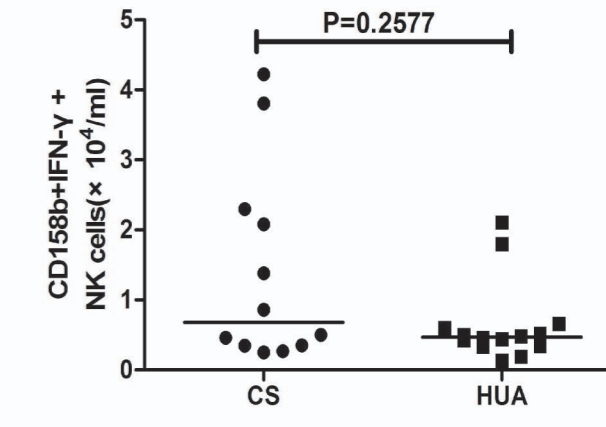

A1

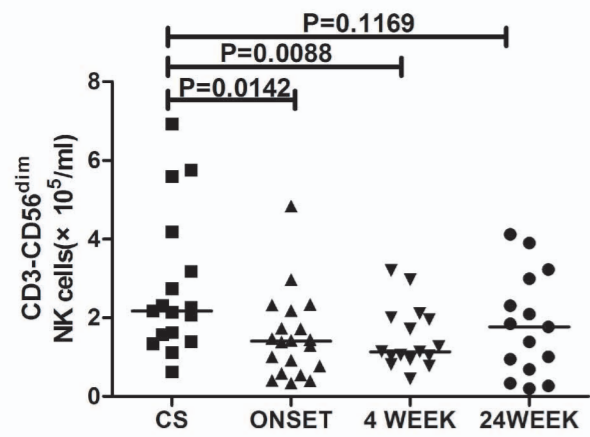

B1

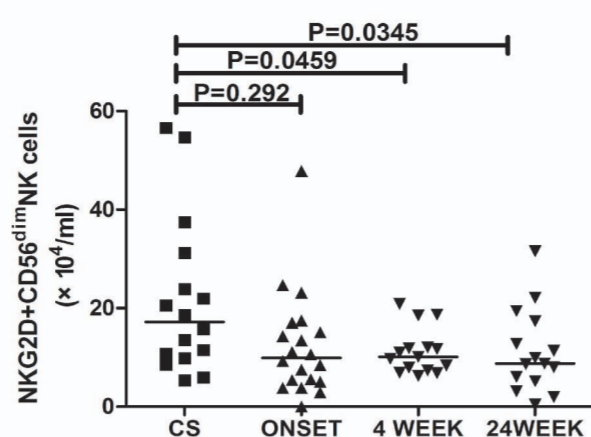

C1

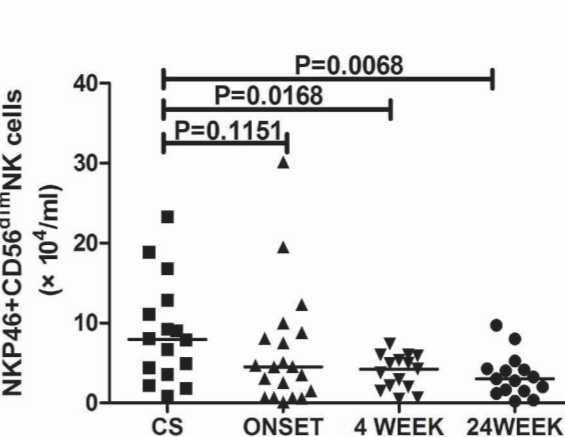

D1

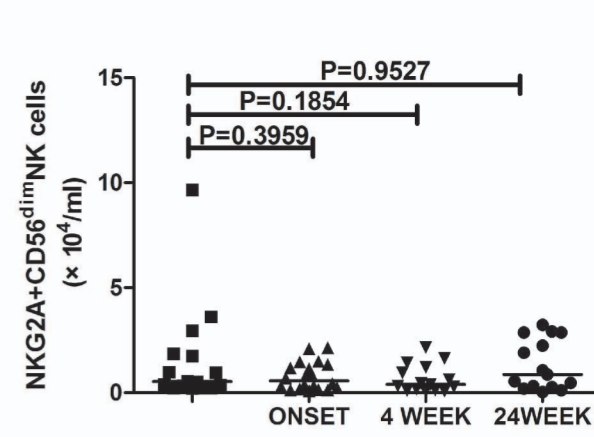

E1

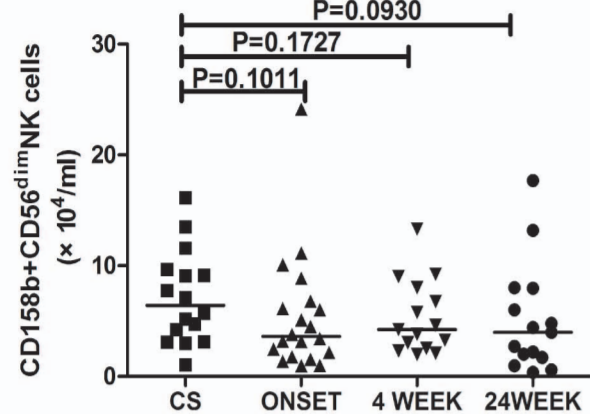

F1

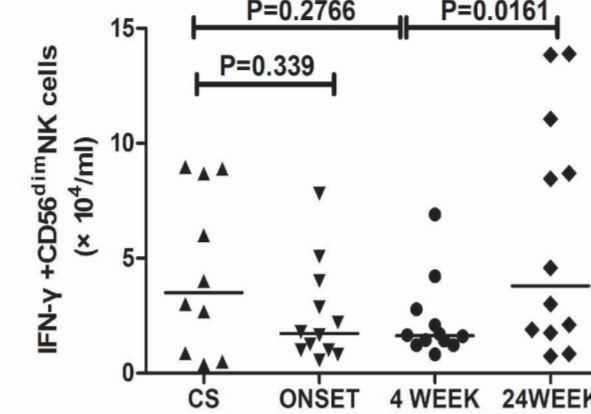

G1

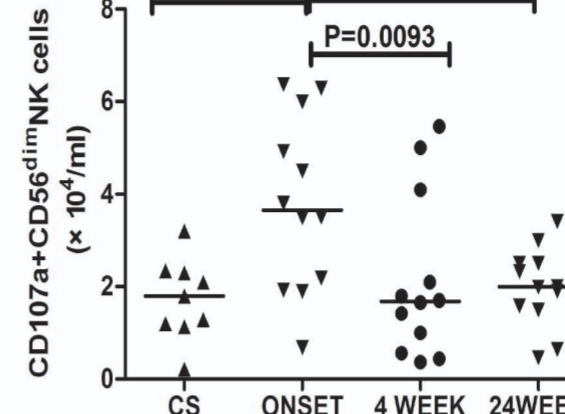

A2

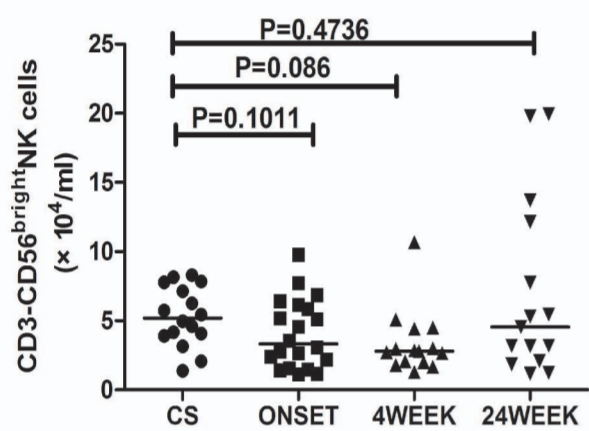

B2

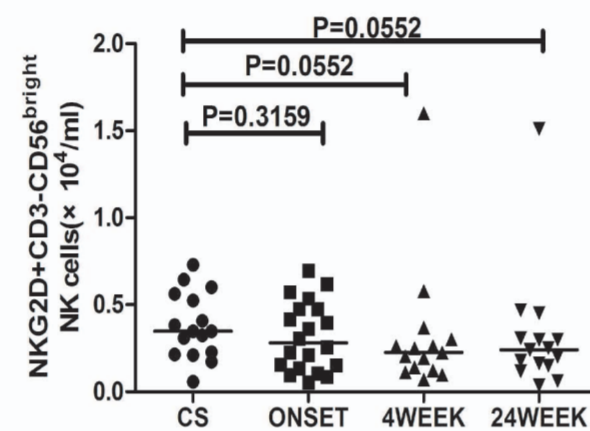

C2

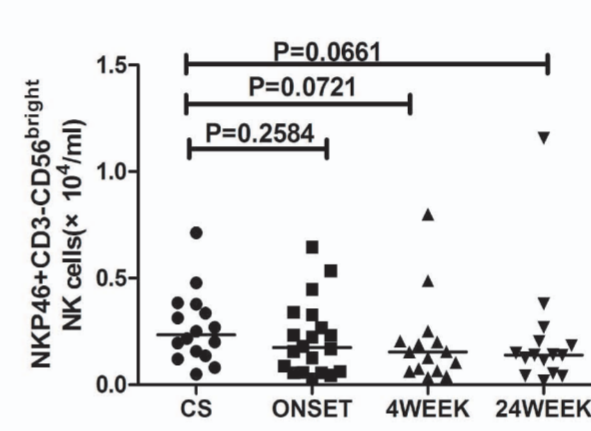

D2

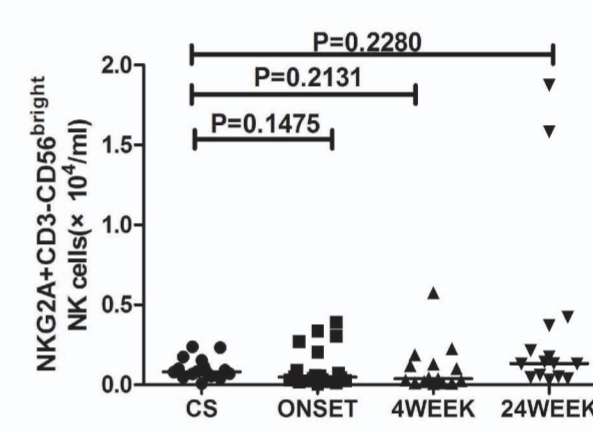

E2

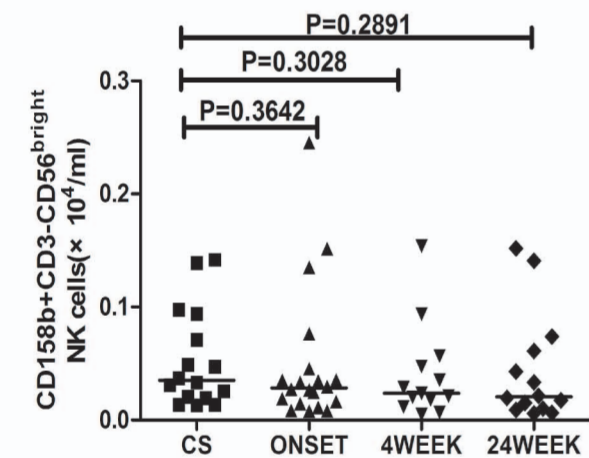

F2

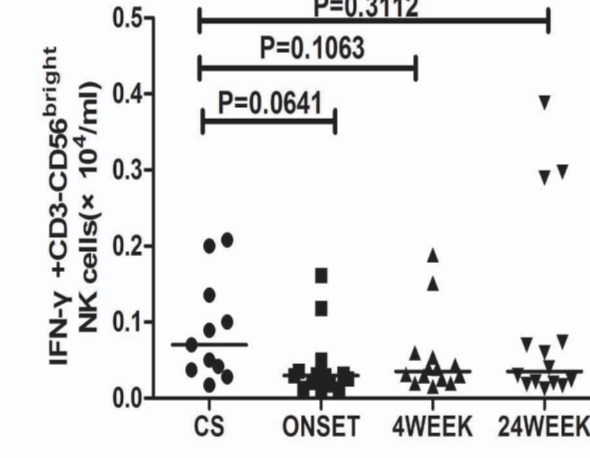

G2

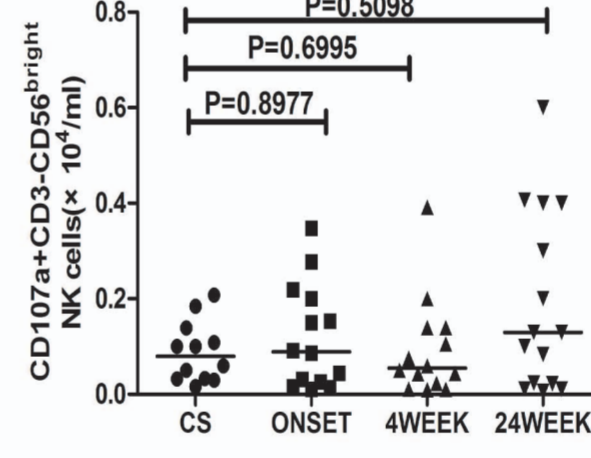

A3

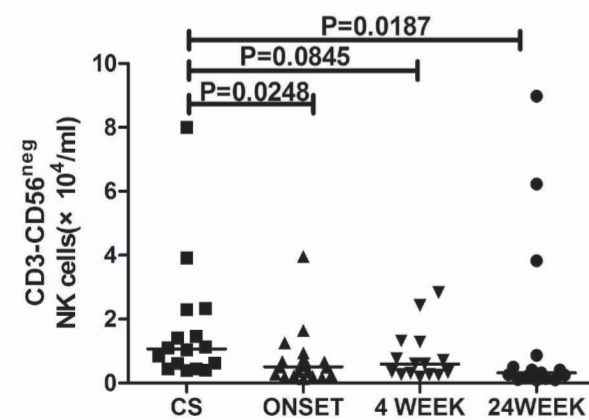

B3

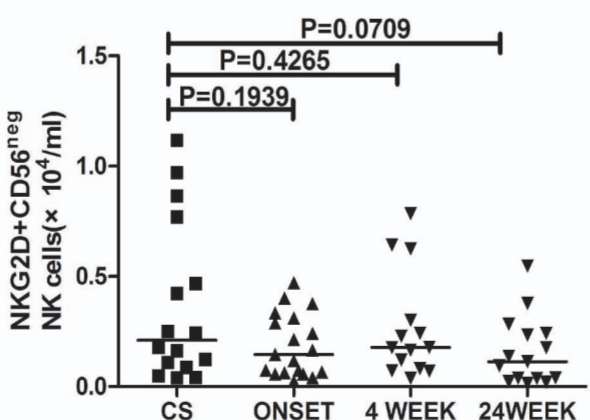

C3

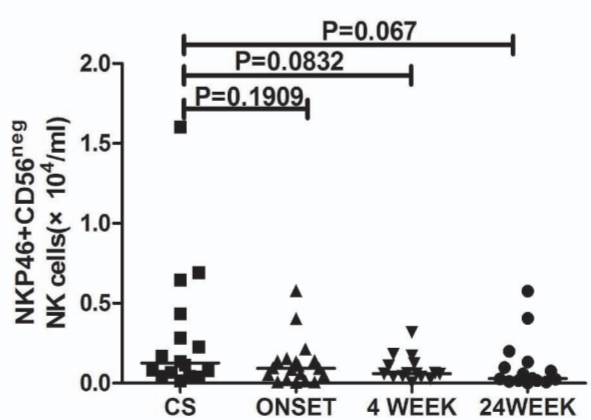

D3

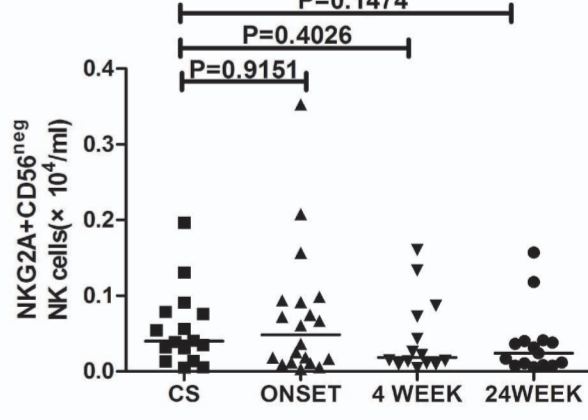

E3

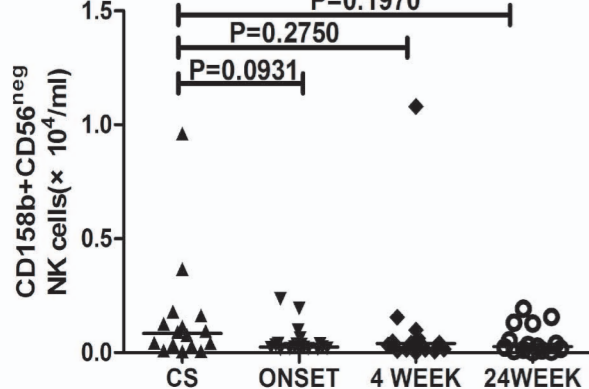

F3

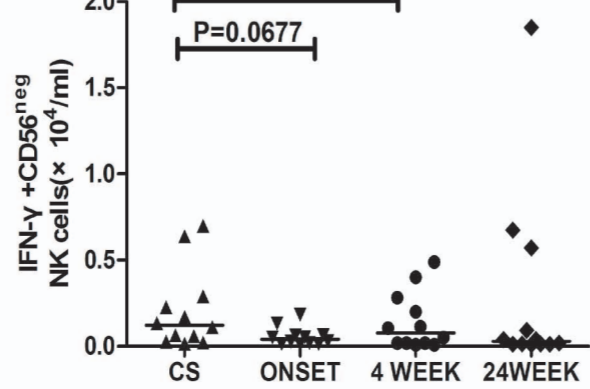

G3

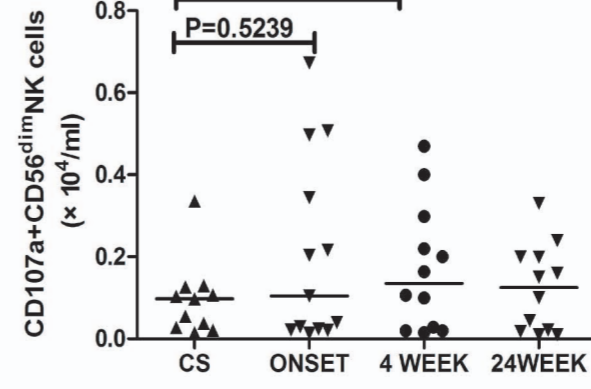

A

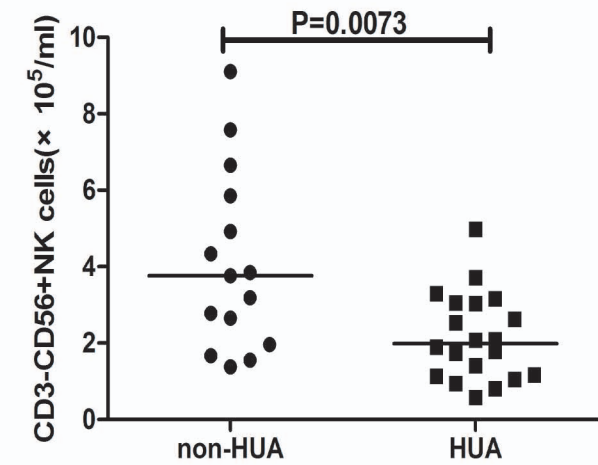

B

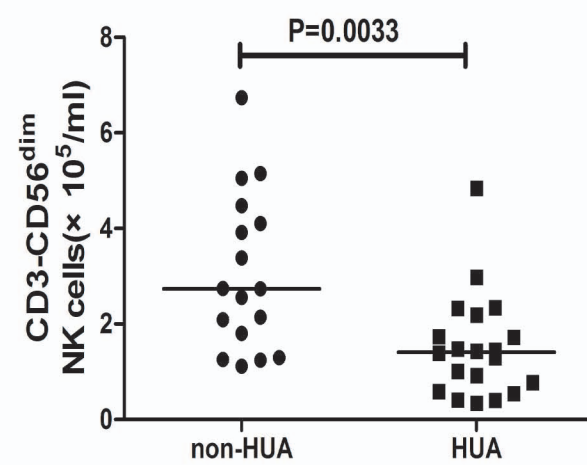

C

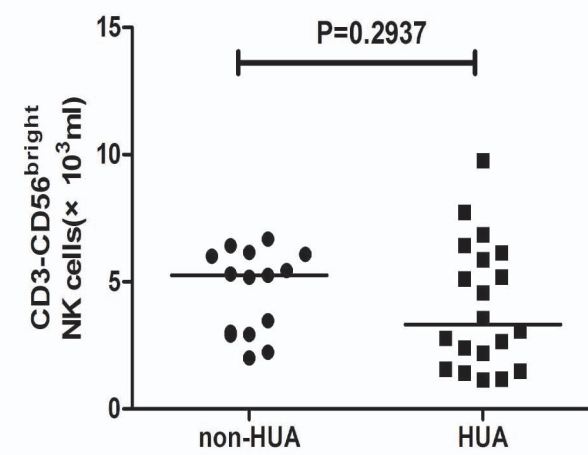

D

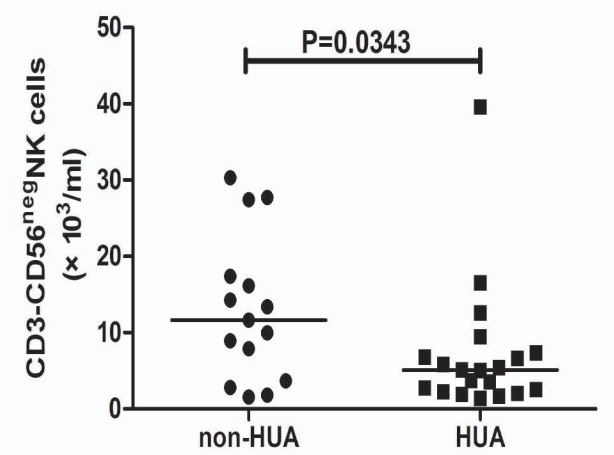

E

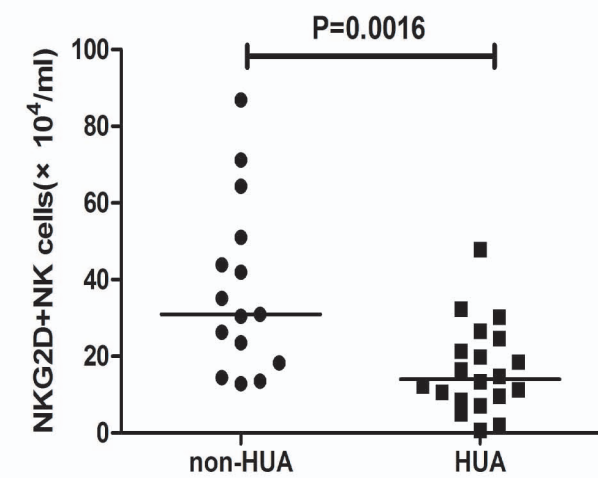

F

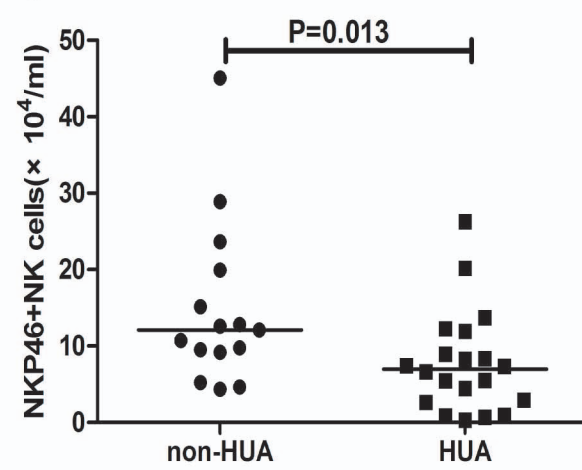

G

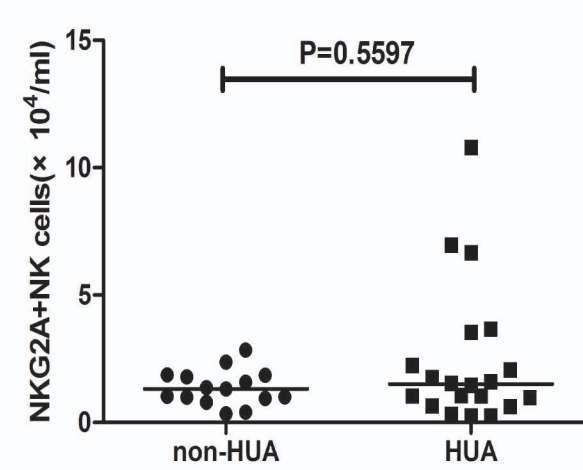

H

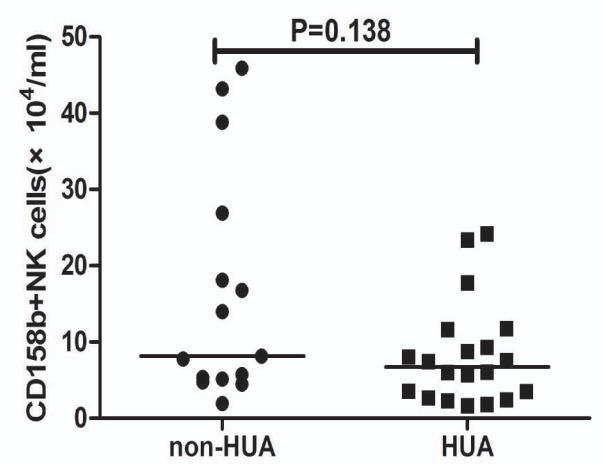

I

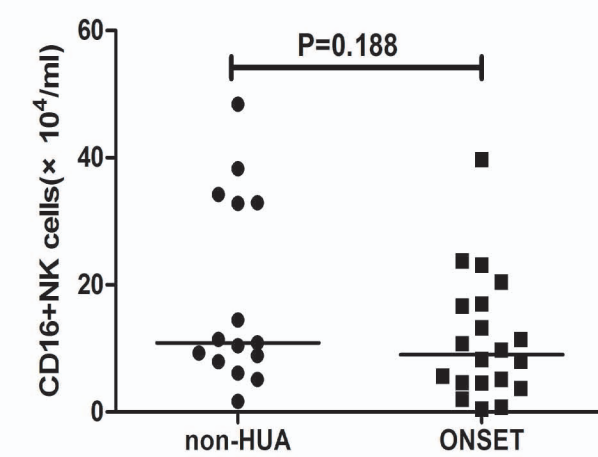

J

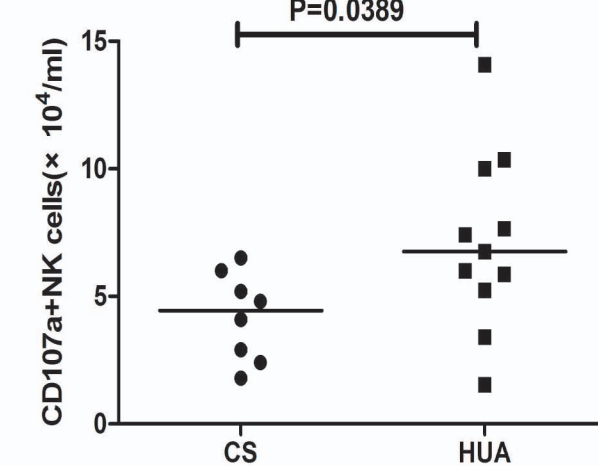

K

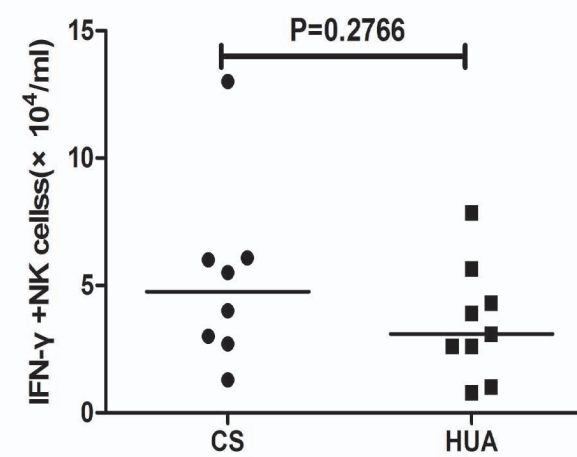

Supplement: Supplemental Digital Content [file medi-97-e13668-s001.pdf]
